# ﻿Fossil Carychiidae (Eupulmonata, Ellobioidea) from the Lower Pleistocene Nashua Formation of Florida, with the description of a new species

**DOI:** 10.3897/zookeys.1167.102840

**Published:** 2023-06-14

**Authors:** Adrienne Jochum, Estée Bochud, David Haberthür, Harry G. Lee, Ruslan Hlushchuk, Roger W. Portell

**Affiliations:** 1 Natural History Museum Bern, Bernastrasse 15, CH-3005 Bern, Switzerland; 2 Institute of Ecology and Evolution, University of Bern, Baltzerstrasse 6, 3012 Bern, Switzerland; 3 Senckenberg Research Institute and Natural History Museum, Senckenberganlage 25, 60325 Frankfurt am Main, Germany; 4 Institute of Anatomy, University of Bern, Baltzerstrasse 2, CH-3012 Bern, Switzerland; 5 Invertebrate Paleontology, Florida Museum of Natural History, 1659 Museum Road, University of Florida, Gainesville, Florida, USA

**Keywords:** Central Florida, computer tomography, freshwater marl, mollusc assemblage, Orlando, Pleistocene

## Abstract

Recent fossil shell mining for a new rail line in the Orlando area of Orange County, Florida has uncovered two species of the ellobioid genus *Carychium* O. F. Müller, 1773 in a bed of freshwater marl from the Lower Pleistocene Nashua Formation. To taxonomically interpret these finds, the well-preserved shells were imaged via high-resolution X-ray tomography (micro-CT) to view significant internal diagnostic characters such as the columellar configuration and the degree of lamellar sinuosity and their relationship in context to the entire shell. The image data are compared to that of type material and extant and fossil *Carychium* species inhabiting the SE USA, Mexico, Central America, and Jamaica. Based on these results, the species *Carychiumfloridanum* G. H. Clapp, 1918 and *Carychiumnashuaense***sp. nov.** are identified from fossil shells dating from the Early Pleistocene. This work documents the first fossil members of *C.floridanum* and the first fossil *Carychium* from the SE USA.

## ﻿Introduction

**Figure 1.**
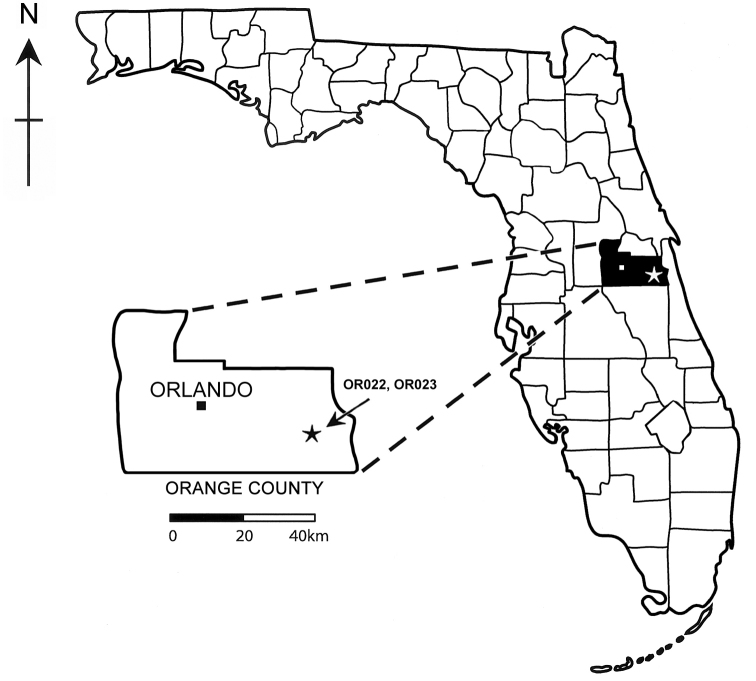
Map showing the location of the stratigraphic sites OR022 and OR023 in Orange County, Florida.

**Figure 2.**
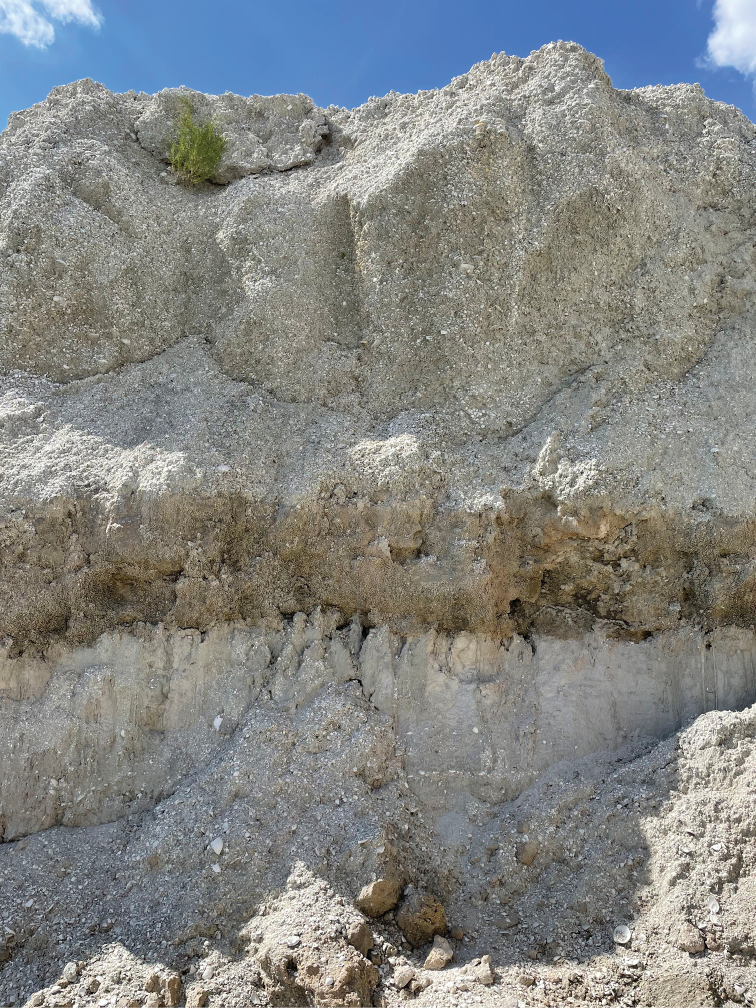
Later stage of excavation showing middle stratum of freshwater marl (c. 1 m thick) wedged between two layers of marine shell layers (each c. 3 m thick).

**Figure 3.**
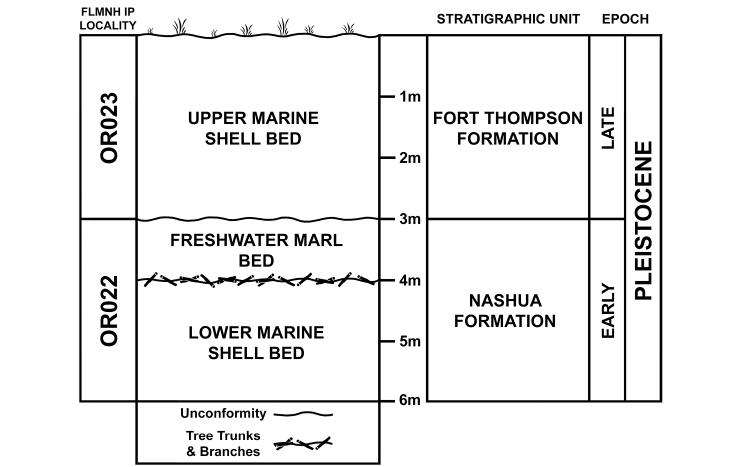
Stratigraphic diagram depicting the distribution of marine shell and freshwater marl layers in the Fort Thompson and Nashua Formation stratigraphic units.

Records of nonmarine fossil molluscs are rare in Florida, and little is known about their distributions in the state. Studies of Pleistocene mollusc faunas in Florida largely focus on marine taxa while fossil nonmarine mollusc beds remain poorly investigated (Auffenberg et al. 2006). During recent shell mining for the track bed of the Brightline Railway connecting Port Canaveral to the Orlando International Airport (Fig. 1), construction engineers serendipitously unearthed a 1 m thick nonmarine lens sandwiched between two marine shell beds. The middle nonmarine lens and the lower marine shell bed date from the Lower Pleistocene Nashua Formation (2.58–0.774 Ma) (Huddlestun 1988) (Figs 2, 3). The upper marine shell layer dates from the Upper Pleistocene Fort Thompson Formation (140–120 ka) (Puri and Vanstrum 1969; Karrow et al. 1996). About 14 freshwater and 28 terrestrial species have so far been identified from this middle layer deposit (to be discussed in a separate work), including well-preserved fossil shells of the terrestrial genus *Carychium* O. F. Müller, 1773.

Though the shells of this malacofaunule will be taxonomically treated and analyzed later in stratigraphic, paleobiogeographic, and ecological contexts, the focus of this present work is on the morphological assessment and taxonomic treatment of the *Carychium* shells. So far, records of fossil *Carychium* are not yet known in Florida. North American Late Cenozoic congeners include fossils of the extant species, *Carychiumexiguum* (Say, 1822) from the Late Pliocene of southwestern Kansas and northern Oklahoma (Hibbard and Taylor 1960) and from the southern High Plains of western Texas and eastern New Mexico (Pierce 1975). Pleistocene deposits of loess and talus are known to contain fossils of *Carychiumexile* H. C. Lea, 1842 in central and eastern Missouri (Greger 1933; Hubricht 1964). Hubricht (1985) also reported the three extant North American *Carychium* species, C.exiguumvar.mexicanumPilsbry (1891), *C.exiguum*, and *C.exile* from Pleistocene fossil material deriving from Texas, Oklahoma, and Kansas. In addition to these North American records, Late Pleistocene fossils of the extant species, *Carychiumjardineanum* (Chitty, 1853), are known from cave deposits in southeast Jamaica (Paul and Donovan 2005).

By using high-resolution X-ray tomography (micro-CT), we compare the internal shell morphology with that of type material of extant congeners found in the SE USA, Mexico, Central America, and Jamaica (Jochum et al. 2017), emphasizing significant diagnostic characters such as the columellar configuration and the degree of lamellar sinuosity and their relationship in context to the entire shell. Hereby, the two southeastern North American species, *Carychiumfloridanum* G. H. Clapp, 1918 and *Carychiumnashuaense* sp. nov., are identified from the Lower Pleistocene Nashua Formation, constituting the first fossils of the genus *Carychium* in the State of Florida and the first *Carychium* fossil record for the SE USA.

## ﻿Material and methods

### ﻿Sampling, imaging, and measurements

Two 7.5 l bulk samples from the middle of the freshwater layer were sorted by hand, washed through a graded series of sieves, and allowed to dry for further processing. Thirty-two *Carychium* shells were culled from a mixture of other molluscs and debris under a stereomicroscope.

All eight shells analyzed in this study are in a good state of preservation and are deposited in the Natural History Museum Bern, Bern, Switzerland (NMBE) and in the Invertebrate Paleontology Division, Florida Museum of Natural History, University of Florida (UF). One shell of *C.floridanum* suffered damage after it was measured and thus, could not be included for the further processing of image data. However, its measurements are included in the measurement data of the fossil material of this species (*N* = 3).

Several qualitative aspects of shell morphology are addressed including peristome shape; whorl profile (whorl convexity); teleoconch sculpture; development of apertural barriers visible in frontal view, including the presence of a deeply immersed denticle/lamella on the parieto-columellar region of the aperture; development of the columellar lamella as discernable in the micro-CT scans of the ventral, dorsal, side-left, side-right and umbilical perspectives of the adult shell.

#### ﻿Light microscopy

Different perspectives of the shells were imaged using a Leica MC190 HD digital camera attached to a Leica M205 stereo microscope (Leica Microsystems GmbH, Wetzlar, Germany). The multifocal images were processed using the software Leica Application Suite X (LAS X) version 5.1.0.25593 (Leica Microsystems). All measurements are in millimeters (mm). Shell measurements are expressed as SH (shell height), SW (shell width), PH (peristome height) and PW (peristome width). All measurements were made using the measuring tool in the Leica LASX application. Shell whorl number was counted (to the closest 0.25 whorl) according to Kerney and Cameron (1979).

#### ﻿X-ray tomographic microscopy (micro-CT)

Aside from the extant *Carychiumfloridanum* shell [NMBE 572256 (ex. AJC 1446)], all scans were processed at the University of Bern, Institute of Anatomy, Bern Switzerland.

The Recent *C.floridanum* shell [NMBE 572256 (ex. AJC 1446)] was imaged using a high-resolution X-ray tomography system (micro-CT), SkyScan 2011 (Bruker micro-CT, Kontich, Belgium) at the Department of Experimental Radiology, Justus-Liebig University Biomedical Research Center Seltersberg (BFS), Giessen, Germany. The shell was mounted and scanned 185° around its vertical axis in rotation steps of 0.23° at 80 kV tube voltage and 120 μA tube current. Reconstruction was performed using the Feldkamp cone beam reconstruction algorithm. Image resolution was 1.75 μm isotropic voxel side length with a grey scale resolution of 8 bit. Digital images post processing and visualization (maximum intensity projection – MIP, volume compositing and summed voxel projection) were displayed using the ANALYZE software package (ANALYZE 11.0, Mayo Clinic, Rochester, MN, USA).

For the fossils in this study, shells were packaged in radiotransparent Basotect melamine resin foam and individually imaged on a Bruker SkyScan 2214 multiscale X-ray micro-computed tomography system at the Institute of Anatomy of the University of Bern in Switzerland (with the control software version 1.8, Bruker micro-CT, Kontich, Belgium). The system is equipped with both a flat-panel detector for scanning large samples and a high-resolution CCD camera, which was used for scanning the *Carychium* shells. The Hamamatsu L10711 X-ray source was set to a tube voltage of 60 kV and a tube current of 130 µA. Individual shells were scanned at a voxel size of 2.0 µm with one shell [*C.floridanum* (NMBE 577015)] at a voxel size of 0.5 µm.

The scans with a resulting voxel size of 2.0 µm were acquired with a set of 1913 projections of 4032 × 2688 pixels at every 0.1° over a sample rotation of 180°. Every single projection was exposed for 2187 min, three projections were averaged to reduce image noise. This resulted in a scan time of approximately 5 h and 15 min.

The scan of *C.floridanum* (NMBE 577015) with a resulting voxel size of 0.5 µm, was acquired with a set of 3601 projections of 4032 × 2688 pixels at every 0.1° over a 360° sample rotation. Every single projection was exposed for 2136 min, three projections were averaged to reduce image noise, which resulted in a scan time of approximately 9 h and 45 min.

The projection images of each scan were reconstructed into a 3D stack of images with NRecon (Version 2.1.0.1, Bruker micro-CT, Kontich Belgium). The entire process resulted in datasets with the isometric voxel size of 2.0 and 0.5 µm, respectively. Scanning parameters are given in Suppl. material 1. The tomographic data was visualized with 3Dscript (Schmid et al. 2019) in Fiji (Schindelin et al. 2012).

### ﻿Repositories

**AJC** Adrienne Jochum Collection: now housed at the NMBE;

**CM**Carnegie Museum of Natural History, Pittsburgh, PA, USA;

**UF**Florida Museum of Natural History, University of Florida, Gainesville, FL, USA;

**NMBE** Natural History Museum Bern, Bern, Switzerland.

## ﻿Results

### ﻿Taxonomy

**Family Carychiidae Jeffreys, 1830**

**Genus *Carychium* O. F. Müller, 1773**

#### ﻿ Carychium floridanum

Taxon classificationAnimaliaEllobiidaEllobiidae

G. H. Clapp, 1918

A5A9E77D-8592-5E97-9C4D-A11BDD6722A1

**Figure 4.**
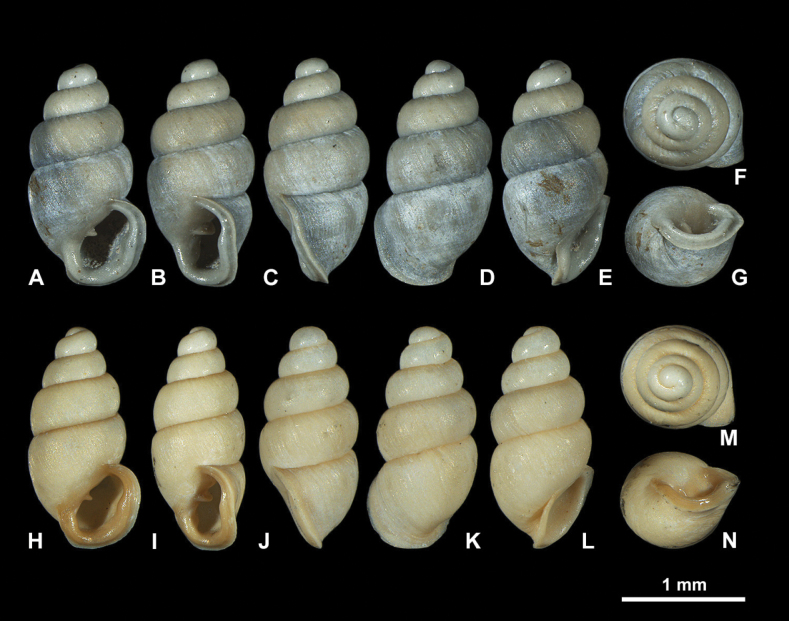
Fossil *Carychiumfloridanum* G. H. Clapp, 1918 (NMBE 577015–577016). Light microscopic images showing different perspectives **A–G** (NMBE 577015) **H–N** (NMBE 577016). Peristome thickly callused with apertural barriers. Grey and orange colours indicate taphonomic and sedimentary processes, repectively. Scale bar: 1 mm.

**Figure 5.**
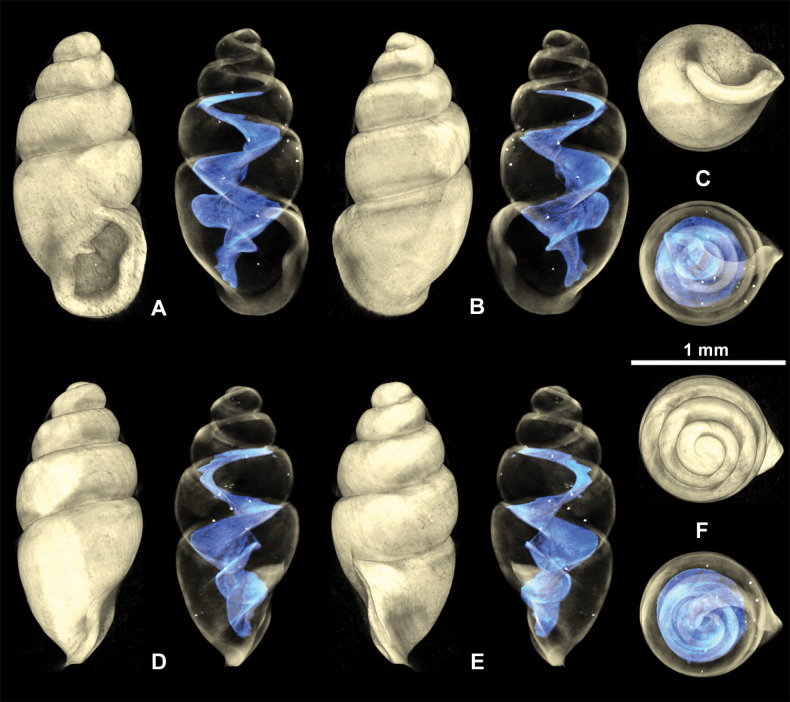
3D visualizations of X-ray micro-CT data of fossil *Carychiumfloridanum* G. H. Clapp, 1918 (NMBE 577015) **A** apertural view showing extent of lamellar fold **B** dorsal view **C** umbilical view showing thick peristome and parietalis **D** aperture facing right view showing tongue-like flexion of the lamella **E** aperture facing left view **F** apical view. Scale bar: 1 mm.

**Figure 6.**
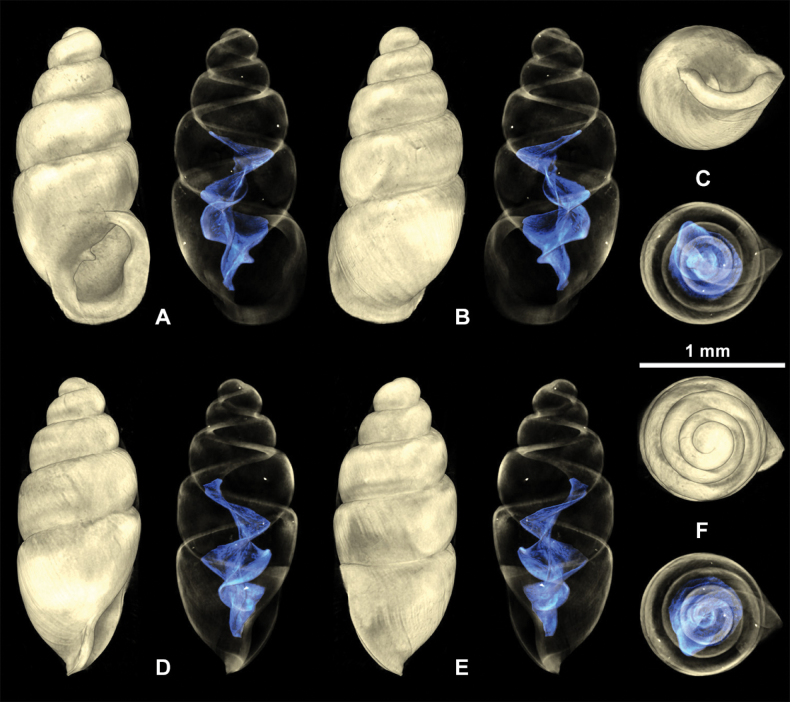
3D visualizations of X-ray micro-CT data of fossil *Carychiumfloridanum* G. H. Clapp, 1918 (NMBE 577016) **A** apertural view **B** dorsal view **C** umbilical view showing thick peristome and parietalis **D** aperture facing right view **E** aperture facing left view **F** apical view. Scale bar: 1mm.

**Figure 7.**
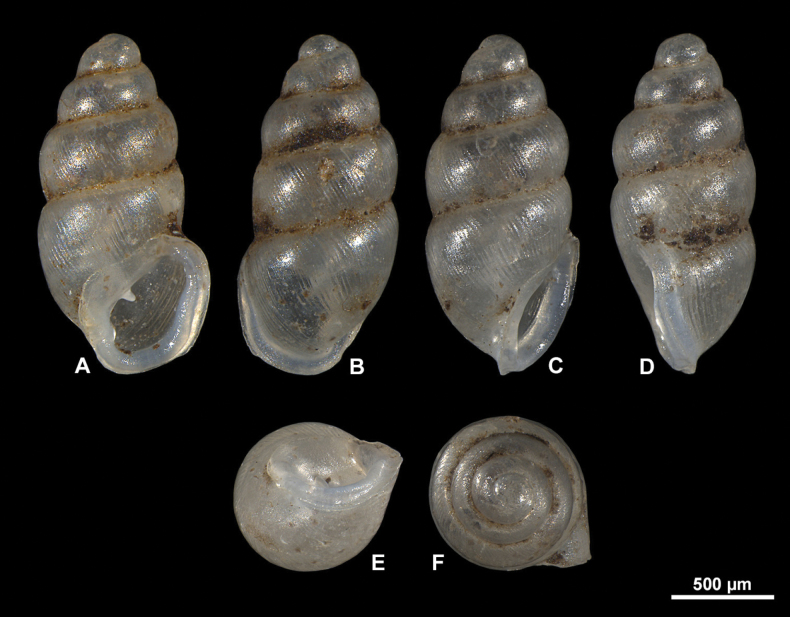
Recent *Carychiumfloridanum* G. H. Clapp, 1918 (NMBE 572256) from Wakulla Springs, Florida. Light microscopic images showing **A** apertural view with thick peristome, parietalis and columellaris **B** dorsal view showing axial ribbing **C** aperture facing right view **D** aperture facing left view **E** umbilical view showing thick peristome and parietalis **F** apical view. Scale bar: 0.5 mm.

**Figure 8.**
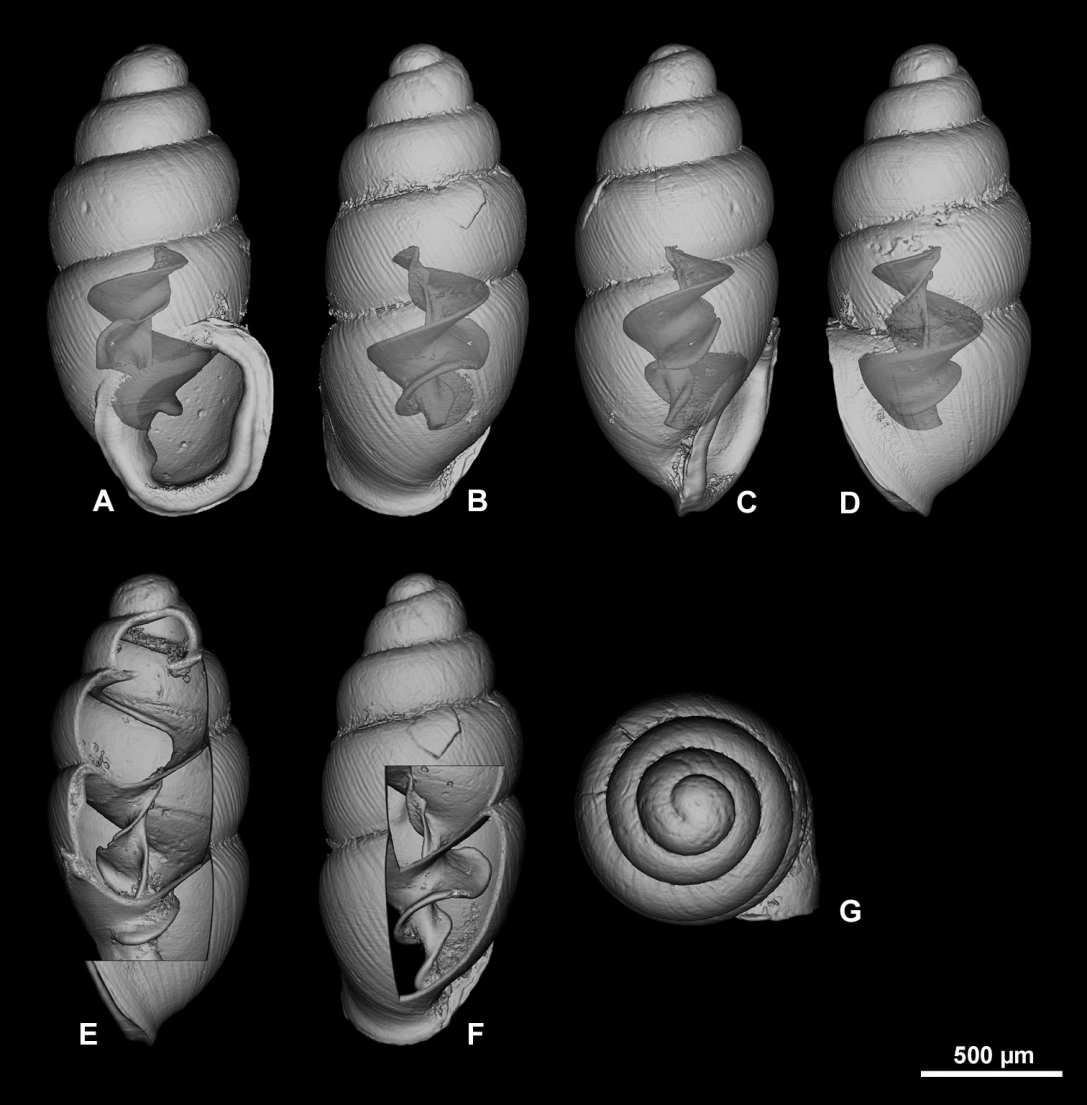
3D visualizations of X-ray micro-CT data of extant *Carychiumfloridanum* G. H. Clapp, 1918 (NMBE 572256) from Wakulla Springs, Florida **A** apertural view **B** dorsal view **C** aperture facing right view **D** aperture facing left view **E** cross section of **D** showing lamellar flexion **F** cross section of **B** showing full columellar extension **G** apical view. Scale bar: 0.5 mm.

Figs 4 , 5 , 6 , 7 , 8

Carychium exiguum floridanum G. H. Clapp, 1918: 73–75, pl. 8.
Carychium floridanum G. H. Clapp, 1918: Weigand et al. 2013: 3, fig. 1 24|C5; Seq. ID: BARCA032-10, BARCA033-10, BARCA034-10, BARCA035-10, BARCA037-10.
Carychium floridanum G. H. Clapp, 1918: Jochum et al. 2017: 110–111, figs 6, 7.

##### Material examined.

USA, Florida • Orange County, Orlando; 28.4489, −81.0375 (WGS84) encompassing 500 m radius; Oct. 2021; R. Portell and H. Means leg.; NMBE 577015–577016 (2 fossil specimens) (Figs 4–6).

The two shells were compared to the syntype shell from Snapper Creek Hammock, Miami, FL (CM 46540) presented in Jochum et al. (2017, figs 6, 7) and additionally, to a Recent shell from the Wakulla Springs, FL population [NMBE 572256 (ex. AJC 1446)] [Weigand et al. 2013, Evolutionary Lineage (EL) C5] (Figs 7, 8).

##### Stratigraphic occurrence.

Freshwater Marl Bed (middle nonmarine layer) Florida Museum of Natural History Invertebrate Paleontology (FLMNH IP) locality OR022, Lower Pleistocene Nashua Formation.

##### Description.

Shell medium-sized for genus (SH: 1.6–1.8 mm), pupiform in shape, glossy; teleoconch with occasional, fine threadlike ribs, with 4.5–5 tumid whorls. Suture deep. Protoconch smooth, bulbous, shiny, and distinct from teleoconch. Whorl profile strongly convex, aperture higher than wide, elliptical, with 2 barriers, parietal robust with the columellaris constituting a low swollen hump deep in the shell. Peristome doubled and heavily callused, expanded in palatal, basal and columellar regions; largely reflected at the base. Palatal lip weakly sigmoid and buttressed with heavy callus inside and marked with a concavity or line at mid-section behind the apertural rim, extending 2/3 the body whorl in aperture facing left profile view (clearly visible on the grey shell and not detectable on the yellowish one) (Fig. 4). PH 38% of SH. The upper palatal part of the peristome is narrower causing the callused mid-section to project beyond the apertural rim (in aperture facing left view). Umbilicus rimate (Figs 5D, 6D). Internally, a single lamella spirals down the columella for at least the last whorl and a half, terminating in the parietalis of the aperture, which is partially visible and positioned near the columellar side of the inner margin of the peristome in umbilical view (Figs 5C, 6C). The lamella is concave, broadly expanded and wing-like, and strongly flexes down immediately before the aperture, forming the robust parietalis (Figs 5A, E, 6A, E). The surface of the lamella is broad (in dorsal view) with the edge sloping slightly oblique to the columella, flipping over forming a thickened, tongue-like structure in aperture facing right view (Figs 5B, D, 6B, D). The early lamella starts tightly spiraling down the columella at about 2/3 the height of the penultimate whorl (Figs 5B, 6B), expanding rapidly to its fullest winged extension in the middle of the penultimate whorl (Figs 5A, 6A). The callused swollen peristome is flush with the convexity of the penultimate whorl in aperture facing left view (Figs 5E, 6E). The regularly coiled spire is broad with the penultimate whorl expanding almost completely over the body whorl in apical view (Figs 5F, 6F).

##### Measurements.

SH = 1.6–1.8 mm; SW = 0.81–0.92 mm; PH = 0.60–0.7 mm; PW = 0.61–0.67 mm (*N* = 3).

##### Occurrence.

A frequent find in the bulk samples of the Freshwater Marl Bed (middle nonmarine layer).

Living *C.floridanum* are known today from central Florida up to Wakulla Springs (Weigand et al. 2013). Earlier collections at the beginning of the 20^th^ century extended as far south as the type locality of Miami in Dade County and in southwestern Florida at Cape Sable, Monroe County (Clapp 1918). Though human encroachment, canalization practices and habitat modification have severely impacted its occurrence in southern Florida, the paucity of recent records in south Florida is likely the result of limited sampling and not extirpation of the species in this part of the state.

##### Recent material for comparison.

*Carychiumfloridanum* G. H. Clapp, 1918, Wakulla Springs, Wakulla County, FL; 30.2355, −84.3031; BARCA032-10; [NMBE 572256 (ex. AJC 1446)] (*N* = 1) (Weigand et al. 2013) (Figs 7, 8).

##### Description and comparison.

Shell pupiform, translucent with fine threadlike ribbing on the teleoconch. 4.5 tumid whorls. Aperture elliptical with 2 barriers; parietalis points downwards and columellaris is stronger than in the fossil shells. The fossil shells show greater whorl convexity and a bulbous protoconch. Peristome unevenly shaped and thickly callused, parietal shield more extensive than in fossil shells. Measurements (NMBE 572256): SH = 1.67 mm; SW= 0.84 mm; PH = 0.68 mm; PW= 0.65 mm.

##### Remarks.

Considering that *C.floridanum* is clearly genetically and morphologically distinct from its SE North American congeners [EL C5 in Weigand et al (2013)], the internal morphology of the northern Wakulla Springs material is congruent with that of the fossil shells here but not fully congruent with that of the southern syntype material (CM 46540) in Jochum et al. (2017). The possibility that the Wakulla Springs population and the fossil shells in this study could instead constitute a northern-central Floridian lineage designated *C.floridanum* by Weigand et al. (2013) has yet to be investigated. Ultimately, until genetic data from *C.floridanum* from southern Florida is available to prove otherwise, we maintain that the fossil and the Wakulla Springs members are *C.floridanum*.

#### ﻿ Carychium nashuaense

Taxon classificationAnimaliaEllobiidaEllobiidae

Jochum, Lee & Portell sp. nov.

55D707AE-931E-5133-A0B1-D955532A82CE

https://zoobank.org/A8021066-EA5D-4152-8E66-9D35C8F0AF69

**Figure 9.**
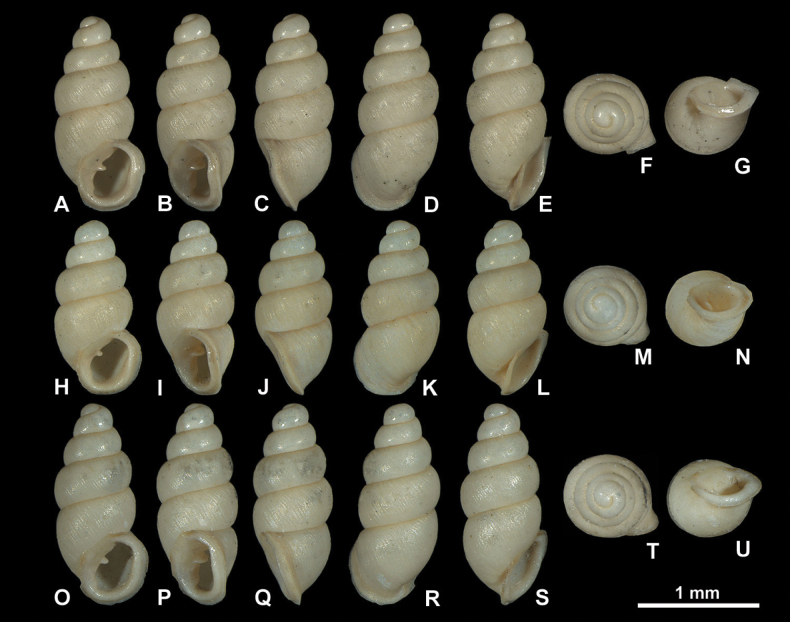
Light microscopic images of *Carychiumnashuaense* sp. nov. **A–G** holotype (NMBE 577017) **H–N** paratype (NMBE 577018) **O–U** paratype (NMBE 577019). Scale bar: 1 mm.

**Figure 10.**
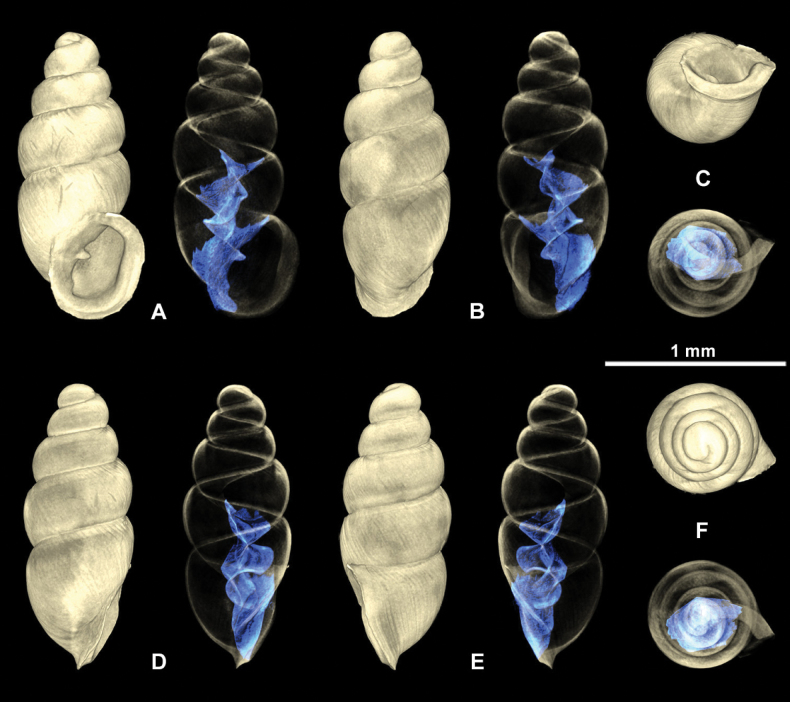
3D visualizations of X-ray micro-CT data of *Carychiumnashuaense* sp. nov. holotype (NMBE 577017) **A** apertural view showing sinuate lamella **B** dorsal view showing entire columellar axis **C** umbilical view showing thick peristome and parietalis **D** aperture facing right view **E** aperture facing left view **F** apical view. Scale bar: 1 mm.

**Figure 11.**
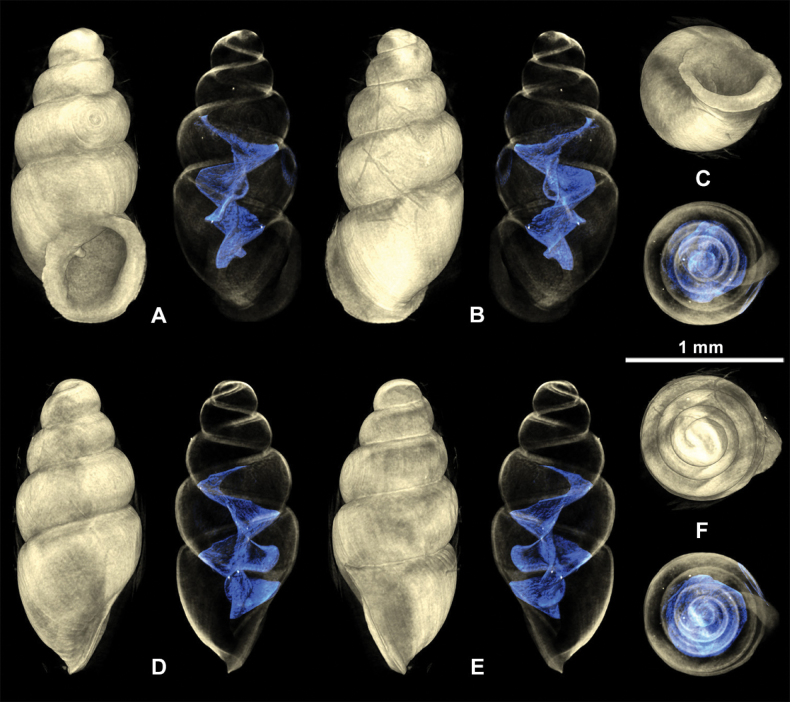
3D visualizations of X-ray micro-CT data of *Carychiumnashuaense* sp. nov. paratype (NMBE 577018) **A** apertural view showing sinuate lamella **B** dorsal view **C** umbilical view showing thick peristome and parietalis **D** aperture facing right view **E** aperture facing left view **F** apical view. Scale bar: 1 mm.

**Figure 12.**
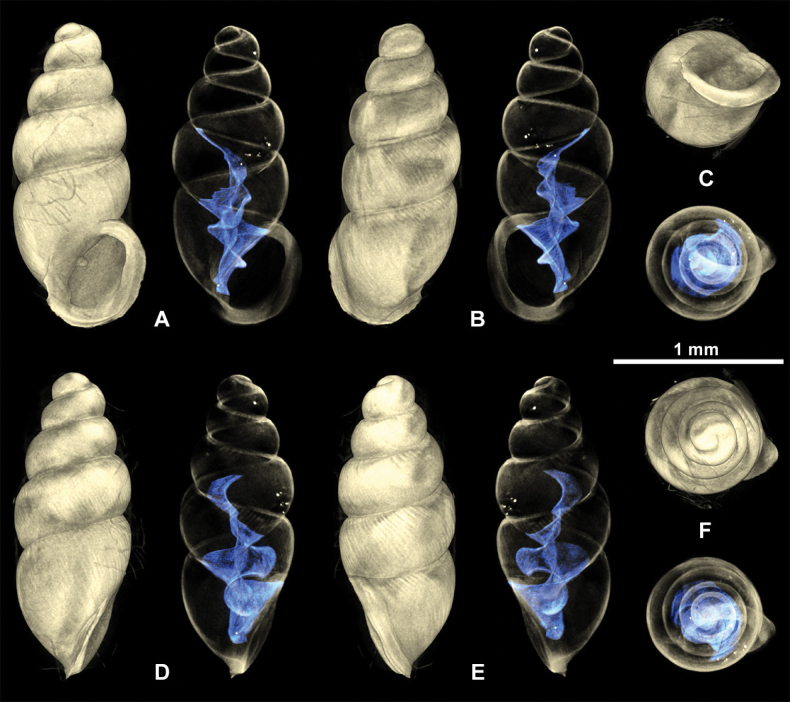
3D visualizations of X-ray micro-CT data of *Carychiumnashuaense* sp. nov. Paratype (NMBE 577019) **A** apertural view showing sinuate lamella **B** dorsal view showing entire columellar axis **C** umbilical view showing thick peristome and parietalis **D** aperture facing right view **E** aperture facing left view **F** apical view. Scale bar: 1 mm.

Figs 9 , 10 , 11 , 12

##### Type material.

***Holotype***: USA, Florida • SH = 1.58 mm, SW = 0.72 mm, PH = 0.56 mm, PW = 0.53 mm; Orange County, Orlando; 28.4489, −81.0375 (WGS84) encompassing 500 m radius; Oct. 2021; R. Portell and H. Means leg.; NMBE 577017. ***Paratypes***: USA, Florida • 2 figured shells; same data as for holotype; NMBE 577018–577019 • 3 shells; same data as for holotype; UF 335884–335886.

##### Measurements.

SH = 1.43–1.63 mm, SW = 0.68–0.69 mm, PH = 0.53–0.57 mm, PW = 0.51–0.54 mm.

##### Diagnosis.

Shell 1.54 mm (mean) in height, elongate-pupiform with elliptical-ovate shaped aperture, thickly callused double peristome with a deeply set columellar-basal apertural barrier and a pronounced parietal denticle. Internally, *C.nashuaense* sp. nov. has a highly sinuate, tightly coiled, double structured lamellar configuration.

##### Description.

Shell medium-sized for the genus, elongate-pupiform, robust, with 5 tumid whorls and a large aperture. PH is 36% of SH, elliptical-ovate, the inner callused, upper palatal side is somewhat angular. Protoconch bulbous, smooth, and shiny; teleoconch ornamented by equidistantly aligned, broad ribs. Suture deeply impressed, not descending towards the aperture. Whorl profile strongly convex, especially on middle whorls, less so on body whorl. Peristome thickly callused and doubled with a thinner, sharp rim at the margins. Columellar portion broadly expanded, heavily callused, palatal lip thicker at mid-section. Outer, basal and columellar margins reflected, parietal callus thick. Two apertural barriers, visible in apertural view; large prominent deeply set parietalis situated almost medially with a slight downward tendency, not reaching margin of peristome; small columellaris near the base of the columella. The moderately thick parietalis is set deep and advances a short distance beyond the thickly callused inner margin of the peristome in umbilical view. Umbilicus chink-like. From apical view, the spire is dominated by the tumid third and fourth whorls causing the body whorl to completely disappear under their convexity. The reflected, wing-like apertural lip projects away from the spire in apical and umbilical view (Figs 9A, 10 C, F). Internally, two lamellae spiral down the columella for at least the last whorl and a half, terminating in the parietalis and columellaris in the aperture. The major lamella is highly sinuate, flipping over in frontal, apertural view (Figs 10A, 11A, 12A). It forms a spatulate, wing-like extension in the penultimate whorl just above the junction with the body whorl in aperture facing right and aperture facing left view (Figs 10D–E, 11D–E, 12D–E). The second, low, tightly coiling lamella proceeds underneath the elaboration of the major lamella, internally forming a conspicuous knob on the columella at mid-section of the body whorl before dropping straight down towards the base of the columella (Figs 10B, 11B, 12B).

##### Differential diagnosis.

*Carychiumnashuaense* sp. nov. appears morphologically to be a cross between *C.belizeense* Jochum & Weigand, 2017 and *C.hardiei* Jochum & Weigand, 2017. These two species are much larger than *C.nashuaense* sp. nov. and have smoother shells. Even though *C.nashuaense* sp. nov. was buried under marl for about 2 million years, the ribbing structure on the teleoconch is remarkably well preserved. The shell shape with the highly convex third and fourth whorls extending over the body whorl, preventing it from being observed in apical view, is most similar to that of *C.belizeense*. On the other hand, the prominent parietalis proceeding far above the peristome edge (umbilical view) and the elliptical apertural form and reduced peristome thickness in that species differs significantly from that of the highly callused *C.nashuaense* sp. nov. Internally, the lamella of C. *belizeense* is highly sinuate and complexly structured on a very compact and short columella.

##### Etymology.

The specific epithet refers to this species being found in the Freshwater Marl Bed of the Nashua Formation.

##### Stratigraphic occurrence.

Freshwater Marl Bed (middle nonmarine layer) (FLMNH IP locality OR022), Lower Pleistocene Nashua Formation.

## ﻿Discussion

The two *Carychium* species of this study show on the one hand, the relative constancy of internal character expression in members of northern and central Floridian *C.floridanum* since the Early Pleistocene and on the other, potential affinity of *C.nashuaense* sp. nov. with a Central American congener for c. 2 million years ago. Though clarified by Jochum et al. (2017), considerations grappled by Clapp (1918), Pilsbry (1948), and Hubricht (1985) concerning the different morphological intergradations (lineages) in *Carychium* across the lower SE USA are still applicable regarding the northern and central Floridian individuals in this study versus the Snapper Creek Hammock syntype of *C.floridanum* in Jochum et al. (2017). Specifically, the reduced expression of characters such as whorl convexity, lamellar configuration, and lack of the parietalis beyond the callused inner margin of the peristome in umbilical view [fig. 7G of *C.floridanum* (CM 46540) in Jochum et al. 2017], introduced new questions beyond the scope of this present work. Future collection from the southernmost region of Florida and DNA analyses of new material may well provide the answers.

Considering the remarkable similarity of *C.nashuaense* sp. nov. with that of *C.hardiei* in Georgia and more so that of *C.belizeense* from Belize in Central America, some parallels can be drawn in conjunction with avian community data from the Plio-Pleistocene in Florida (Emslie 1998).

Birds are considered to have facilitated long distance dispersal in snails and as a result, their distributions. They are known vectors of expanded snail distributions via snail transport in the gut and in their plumage (Green and Figuerola 2005; Kappes and Haase 2012; van Leeuwen and van der Velde 2012; van Leeuwen et al. 2012, 2013). *Carychium* species live in wetlands where they share the same habitats with transient bird populations. During the Plio-Pleistocene, range expansions of Neotropical, western North American, and continental forest birds into Florida are known to have occurred during glacial stages (Emslie 1998). The rapid climate cycles influenced terrestrial community structure with the formation and fragmentation of the Gulf Coast corridor, which joined Neotropical regions to the south with western North America and the Florida peninsula (Emslie 1998). The exposure of the shallow continental shelf in the Gulf of Mexico during the glacial stages greatly expanded the Floridian peninsular land mass, allowing several species of mammals, birds, reptiles, and plants to extend their ranges into Florida. *Carychium* from Central American and Caribbean populations could have easily been transported by these new immigrants into the wetland habitats from which the washed-in sediments of the Freshwater Marl Bed derived. The introduction of congeners from the north and south and the subsequent intermixing of species would have provided new opportunity for new species such as *C.nashuaense* sp. nov. and the north-central Floridian *C.floridanum* lineage C5 (Weigand et al. 2013) to evolve.

Given the fossil site’s close proximity (less than 50 km) to the coast of the Atlantic Ocean and its surrounding low-lying topography (< 21 m at the surface), the area has been subject to transgressive (landward migration of the shoreline) and regressive (oceanward migration of the shoreline) episodes due to sea-level oscillations during the Pleistocene (Hine 2013). Therefore, the two marine deposits accumulated in the area during separate transgressive cycles and the freshwater marl deposit (with washed-in terrestrial constituents) accumulated in either freshwater ponds or swamps of low-lying coastal areas.

We strongly encourage continued exploration and investigations in other regions of Florida to glean more knowledge about *Carychium* distribution, ecology, and community structure in the state.

## Supplementary Material

XML Treatment for Carychium floridanum

XML Treatment for Carychium nashuaense

## ﻿Additional information

### Author ORCIDs

Adrienne Jochum https://orcid.org/0000-0002-6624-6412

David Haberthür https://orcid.org/0000-0003-3388-9187

### Data availability

All of the data that support the findings of this study are available in the main text or Supplementary Information.

## Supplementary materials

Supplementary material 1 X-ray micro-CT parameters Data type micro-CT data (.xlsx file) https://binary.pensoft.net/file/864004This dataset is made available under the Open Database License (http://opendatacommons.org/licenses/odbl/1.0/). The Open Database License (ODbL) is a license agreement intended to allow users to freely share, modify, and use this Dataset while maintaining this same freedom for others, provided that the original source and author(s) are credited.Adrienne Jochum, Estee Bochud, David Haberthür, Harry G. Lee, Ruslan Hlushchuk, Roger W. Portell
